# Prediction of protein structural classes for low-homology sequences based on predicted secondary structure

**DOI:** 10.1186/1471-2105-11-S1-S9

**Published:** 2010-01-18

**Authors:** Jian-Yi Yang, Zhen-Ling Peng, Xin Chen

**Affiliations:** 1 Division of Mathematical Sciences, School of Physical and Mathematical Sciences, Nanyang Technological University, 21 Nanyang Link, Singapore, 637371; 2 Department of Mathematics, Bijie University, Guizhou, PR China, 551700

## Abstract

**Background:**

Prediction of protein structural classes (*α*, *β*, *α *+ *β *and *α*/*β*) from amino acid sequences is of great importance, as it is beneficial to study protein function, regulation and interactions. Many methods have been developed for high-homology protein sequences, and the prediction accuracies can achieve up to 90%. However, for low-homology sequences whose average pairwise sequence identity lies between 20% and 40%, they perform relatively poorly, yielding the prediction accuracy often below 60%.

**Results:**

We propose a new method to predict protein structural classes on the basis of features extracted from the predicted secondary structures of proteins rather than directly from their amino acid sequences. It first uses PSIPRED to predict the secondary structure for each protein sequence. Then, the *chaos game representation *is employed to represent the predicted secondary structure as two time series, from which we generate a comprehensive set of 24 features using *recurrence quantification analysis*, *K-string based information entropy *and *segment-based analysis*. The resulting feature vectors are finally fed into a simple yet powerful Fisher's discriminant algorithm for the prediction of protein structural classes. We tested the proposed method on three benchmark datasets in low homology and achieved the overall prediction accuracies of 82.9%, 83.1% and 81.3%, respectively. Comparisons with ten existing methods showed that our method consistently performs better for all the tested datasets and the overall accuracy improvements range from 2.3% to 27.5%. A web server that implements the proposed method is freely available at http://www1.spms.ntu.edu.sg/~chenxin/RKS_PPSC/.

**Conclusion:**

The high prediction accuracy achieved by our proposed method is attributed to the design of a comprehensive feature set on the predicted secondary structure sequences, which is capable of characterizing the sequence order information, local interactions of the secondary structural elements, and spacial arrangements of *α *helices and *β *strands. Thus, it is a valuable method to predict protein structural classes particularly for low-homology amino acid sequences.

## Background

The biological function of a protein is essentially associated with its tertiary structure, which is believed to be determined by its amino acid sequence via the process of protein folding [1]. Therefore, the prediction of protein's tertiary structure from amino acid sequences is a very important while challenging task in computational biology and proteomics. The tertiary structure can be broadly categorized into four structural classes based on the types and arrangements of their secondary structural elements [2]. They are the *α *class in which proteins contain mainly helices, the *β *class containing mainly strands, and the other two classes with a mixture of *α *helices and *β *strands - the *α *+ *β *class having *β *strands mainly antiparallel and the *α*/*β *class having *β *strands mainly parallel. It is of great value to predict protein structural classes as it is beneficial to study protein function, regulation and interactions. For instance, the searching scope of conformation will be significantly reduced for proteins whose structural classes are known [3].

A number of methods have been proposed to predict protein structural classes from amino acid sequences [4-11]. They mainly differ in the selection of feature sets used for prediction. The most common features are on the basis of the amino acid composition, which generally represent a protein as a twenty-dimensional vector corresponding to the frequencies of twenty amino acids in a given protein amino acid sequence [4,7,8]. However, these features ignored the important sequence order information which has been shown beneficial to the predictions. To overcome this limitation, various new features were developed on the basis of a so-called *pseudo amino acid (PseAA) composition *[12], and have been shown very successful in the prediction of protein structural classes [13,14], especially for high-homology protein datasets. However, when low-homology datasets with pairwise sequence identity below 40% were tested, these methods were not effective any more. For instance, for the widely used dataset *25PDB *whose sequence homology is about 25%, the reported overall accuracies with these methods were about 60% only [5,6]. Recently, Kurgan *et al. *[15,16] proposed to extract features from the predicted secondary structure content rather than directly from the protein's amino acid sequence, and reported that the higher prediction accuracy can be consequently achieved, for instance, the overall accuracy of 79.7% for the dataset *25PDB *[16].

In this study, we would like to introduce a new comprehensive feature set that was also constructed from the predicted secondary structure, and demonstrate by experiments on three benchmark datasets that the prediction of protein structural classes can be further improved for low-homology amino acid sequences.

## Results and Discussion

### The proposed method

In the first step, we use the tool PSIPRED to predict the protein secondary structure for an amino acid sequence of interest. Then, the *chaos game representation *is employed to represent a predicted secondary structure as two time series, from which we generate a comprehensive set of 24 features using *recurrence quantification analysis*, *K-string based information entropy *and *segment-based analysis*. The recurrence quantification analysis aims to capture the sequence order information of the time series [17], the *K*-string based information entropy to reflect certain local interactions along the secondary structure [18], and the segment-based analysis to characterize the spacial arrangements of *α *helices and *β *strands (which is mainly used to differentiate between the *α *+ *β *and *α*/*β *classes). Finally, the resulting 24-dimensional feature vector is fed into a simple yet powerful Fisher's discriminant algorithm [19] to make prediction of its protein structural class. Please see the section **Methods **for the details on the feature construction. A web server that implements the proposed method is freely available at [20].

### Prediction accuracies for three benchmark datasets

The proposed method is tested on three benchmark datasets in low homology, including *25PDB *that comprises 1673 proteins of about 25% sequence identity, *640 *that comprises 640 proteins of about 25% sequence identity and *1189 *that comprises 1092 proteins of about 40% sequence identity. The resulting prediction accuracies are listed in Table 1. It can be seen that the overall accuracies for the three datasets are all above 80%. To be specific, the overall accuracies of 82.9%, 83.1% and 81.3% are achieved for the datasets *25PDB*, *640 *and *1189*, respectively. If comparing the four structural classes to each other, the predictions of proteins in the *α *classes are always the best (with accuracies about 90% for all the datasets).

**Table 1:** Prediction accuracies of our method for three datasets and comparison with other reported results.

Dataset	Reference	Prediction accuracy (%)				
		*α*	*β*	*α *+ *β*	*α*/*β*	Overall
*25PDB*	[6]	69.1	61.6	60.1	38.3	57.1
	[22]	60.6	60.7	44.3	67.9	58.6
	[5]	NA	NA	NA	NA	59.9
	[15]	77.4	66.4	45.4	61.3	62.7
	[17]	64.3	65.0	61.7	65.0	64.0
	[16]	**93.7**	81.3	**71.7**	73.1	80.3
	This paper	92.8	**83.3**	70.1	**85.8**	**82.9**
*640*	[21]	73.9	61.0	33.9	81.9	62.3
	[16]	**90.6**	81.8	66.7	85.9	80.8
	This paper	89.1	**85.1**	**71.4**	**88.1**	**83.1**
*1189*	[7]	NA	NA	NA	NA	53.8
	[6]	57.0	62.9	25.3	64.6	53.9
	[23]	NA	NA	NA	NA	54.7
	[14]	48.9	59.5	26.6	81.7	56.9
	[5]	NA	NA	NA	NA	58.9
	[17]	62.3	67.7	63.1	66.5	65.2
	[21]	75.8	75.2	31.8	82.6	67.6
	[16]	87.4	84.7	53.1	**84.7**	78.3
	This paper	**89.2**	**86.7**	**65.6**	82.6	**81.3**

The accuracies are evaluated by jackknife test and measured by the percentage of correctly predicted Proteins. The best results are highlighted in bold face.

We also obtained satisfactory prediction accuracies (about 85%) for proteins in the *β *and *α*/*β *classes. However, it seems very challenging to predict proteins in *α *+ *β *classes as their prediction accuracies are relatively low (ranging between 65.6% and 71.4%) when compared with the other classes. As a previous study pointed out [21], the low prediction accuracy of the *α *+ *β *class might be due to its non-negligible overlap with the other classes.

### Comparison with existing methods

The proposed method were compared with ten existing methods [5-7,14-17,21-23], and the experimental results are listed in Table 1. Except for the method *SCPRED *[16], the listed accuracy values are taken directly from their respective references. Because some inconsistencies were found between our test dataset *25PDB *and the one used in [16] to test *SCPRED *(see **Methods**), the direct comparison with the accuracy values reported in [16] would not be fair. Therefore, to ensure a fair comparison, we re-implemented the method *SCPRED *by following the details presented in the reference paper, trained its classifier with the same version of PSIPRED used for our method, and then applied it to our test dataset *25PDB*. It turns out that, the obtained accuracy values (by the jackknife test) for predicting proteins in the *α*, *β*, *α *+ *β *and *α*/*β *classes are 93.7%, 81.3%, 71.7% and 73.1%, respectively, giving rise to the improvements of 1.1% 1.2% and 0.7% for the first three classes over those given in the reference paper. The overall prediction accuracy hence increases by 0.6% with our test dataset as well. These new accuracy values are listed in Table 1, and we use them as the performance measurements of the method *SCPRED *for comparison.

From Table 1, we can see that the proposed method achieved the highest overall prediction accuracies among all the tested methods. By compared to the second highest accuracy values that were obtained with the method *SCPRED*, there are improvements of 2.6%, 2.3%, and 3% for the three test datasets, respectively. We also notice that significant improvements were made in particular for the *α *+ *β *class and the *α*/*β *class. For example, the proposed method obtained the 85.8% accuracy for predicting proteins of the *α*/*β *class from the dataset *25PDB*, which is 12.7% higher than that given by the method *SCPRED*. When the dataset *1189 *is tested, the accuracy for predicting proteins of the *α *+ *β *class is 12.5% higher than that given by the method *SCPRED*. Bear in mind that both *SCPRED *and our proposed method use features that are extracted from the secondary structure predicted with PSIPRED. The prediction improvements hence clearly indicate that our features are more comprehensive and informative than those used by *SCPRED*.

### Contribution of features

To represent a protein, we used three different approaches to extract features from the predicted secondary structure sequences -- recurrence quantification analysis, *K*-string based information entropy, and segment-based analysis. For brevity, let *R*, *K *and *S *denote the feature subsets generated by these three approaches, respectively. Below, we investigate how these feature subsets contribute to the prediction results.

Table 2 lists the overall prediction accuracies that were obtained with all the possible combinations of feature subsets. It can be seen that when the feature subsets are used individually, the resulting overall prediction accuracies for three datasets are all well above 25%. It indicates that these predictions are unlikely to be random, since random assignment of protein classes generally leads to an accuracy value of about 25%. In other words, every feature subset makes its own positive contributions to the predictions. On the other hand, as more features are involved in the prediction, the overall accuracy values are shown to increase steadily (The only exceptional case occurs when the feature subset *K *is combined with the feature subset *R*, in which the accuracy value decreases slightly from 81.4% to 81.2%). For instance, when tested on the dataset *640*, the prediction accuracy with the feature subset *R *is 80.5%. If the feature subset *K *is added, the accuracy value increases to 81.1%. If the feature subset *S *is further added, i.e., all the extracted features are used, the accuracy value increases by another 2.0% up to 83.1%. Therefore, we may conclude that these three feature subsets can make complementary contributions to each other to the predictions of protein structural classes.

**Table 2:** Overall accuracies obtained with different combinations of feature subsets.

Dataset	*R*	*K*	*S*	*R *+ *K*	*R *+ *S*	*K *+ *S*	*R *+ *K *+ *S*
*25PDB*	81.4	76.0	72.7	81.2	82.3	78.2	**82.9**
*640*	80.5	77.2	73.9	81.1	82.5	78.9	**83.1**
*1189*	79.6	75.8	73.0	80.3	81.0	79.7	**81.3**

See text for the notations of *R*, *K *and *S*.

### Differentiating between the *α *+ *β *and *α*/*β *classes

Because the segment-based features (i.e., the feature subset *S*) are aimed to mainly differentiate between the *α *+ *β *and *α*/*β *classes, it is very interesting to know how effective they are. To avoid any potential outside effects, we would like to make tests on (pure) datasets that comprise proteins only from the *α *+ *β *and *α*/*β *classes. For this purpose, we generate a subset for each benchmark dataset by removing all the proteins in the *α *class or the *β *class, and then train the classifier (i.e., Fisher's discriminant algorithm in our study) on these reduced subsets instead of the whole datasets.

Table 3 lists the prediction accuracy values obtained with the reduced subsets using different combinations of feature subsets. As we can see from the table, the combination *R *+ *K *provides the overall prediction accuracies that are only comparable to those given by the method *SCPRED*. In particular for the dataset *640*, it even gives a lower accuracy value (82.2% v.s. 83.3%). With the addition of the feature subset *S*, the overall prediction accuracies got improved by about 3.0%, and most importantly, all exceed those given by the method *SCPRED*. Specifically, there are the accuracy improvements of 4.3%, 2.6%, and 4.9% for the three test datasets, respectively. These experiments further demonstrate that the segment-based features are very valuable for differentiating between the *α *+ *β *and *α*/*β *classes.

**Table 3:** The accuracies of differentiating between the *α *+ *β *and *α*/*β *classes.

	*R *+ *K*			*R *+ *K *+ *S*			**Ref. **[16]		
			
Dataset	*α *+ *β*	*α*/*β*	Overall	*α *+ *β*	*α*/*β*	Overall	*α *+ *β*	*α*/*β*	Overall
*25PDB*	79.1	84.4	81.4	82.8	86.4	**84.4**	83.2	76.0	80.1
*640*	78.4	85.9	82.2	83.6	88.1	**85.9**	77.2	89.3	83.3
*1189*	76.8	83.2	80.5	81.3	83.8	**82.8**	63.1	88.6	77.9

The datasets comprise only proteins in the *α *+ *β *and *α*/*β *classes.

## Conclusion

To predict structural classes for low-homology protein sequences for which the pairwise sequence identity lies between 20% and 40%, existing methods work very poorly with only relatively low accuracies obtained. In this paper, we aim to develop a new method so as to improve the prediction accuracy. To do so, we first use PSIPRED to predict the secondary structure sequence from a given amino acid sequence. Then, the chaos game representation (CGR) is employed to represent the predicted secondary structure as two time series, from which a comprehensive set of 24 features are generated by three different approaches -- that is, the recurrence quantification analysis, *K*-string based information entropy, and segment-based analysis. The resulting feature vectors, each representing one protein, are fed into Fisher's discriminant algorithm for the final prediction of protein structural classes. Experimental results showed that all these features can make their own positive and complementary contributions so that higher prediction accuracies are achieved. For example, to predict structural classes of proteins in the dataset *25PDB*, it achieved the accuracies of 92.8%, 83.3%, 70.1% and 85.8% for the *α*, *β*, *α *+ *β *and *α*/*β *classes, respectively, and the overall accuracy of 82.9%, which is 2.6% higher than that given by the state-of-the-art method *SCPRED*.

By comparisons with ten existing methods, we may attribute the high prediction accuracy of the proposed method to the superior performance of PSIPRED in predicting secondary structures and the comprehensive set of features that we constructed. The first attribution can be seen from the comparison with the method proposed in [15], which used the secondary structure prediction tool developed in [24] instead of PSIPRED. A previous study [25] showed that PSIPRED is superior to other competing secondary structure prediction methods. The second attribution can be seen from the comparison with the method *SCPRED*, which differs from our proposed method mainly in the selection of features. We used three different approaches to extract a comprehensive set of features from the predicted secondary structures, where the recurrence quantification analysis is used to capture the sequence order information of the time series, the *K*-string based information entropy to reflect certain local interactions along the secondary structure, and the segment-based features to characterize the spacial arrangements of *α *helices and *β *strands. Thus, our proposed method may provide a promising tool for the accurate prediction of protein structural classes, in particular for low-homology amino acid sequences.

## Methods

### Datasets

The proposed method is tested on three low-homology protein datasets that are widely used in the literature, and compared to a variety of existing methods [5-7,14-17,21-23]. The first two datasets, referred to as *25PDB *and *1189 *respectively, are downloaded from RCSB Protein Data Bank [26] with the PDB IDs listed in the paper [6]. The dataset *25PDB *contains 1673 proteins of pairwise sequence identity being about 25%, whereas the dataset *1189 *contains 1092 proteins of 40% sequence identity. The third protein dataset, referred to as *640*, was first studied in [21]. It contains 640 proteins of 25% sequence identity and freely available from the web server at [27]. Note that the amino acid sequences in these datasets indeed represent protein domains rather than the complete proteins. Table 4 lists the numbers of proteins belonging to each structure class for the above three datasets, where protein structural classifications are retrieved from the database SCOP [28] and considered as *true *for prediction evaluation.

**Table 4:** The number of proteins belonging to different structural classes in the datasets.

Dataset	*α*	*β*	*α*/*β*	*α *+ *β*	Total
*25PDB*	443	443	346	441	1673
*640*	138	154	177	171	640
*1189*	223	294	334	241	1092

It shall be mentioned that protein sequences of the *25PDB *dataset are also provided at [29] by the study of [16]. However, some of them are different from those in our test dataset that were instead downloaded from the RCSB Protein Data Bank, which would not allow for a fair performance comparison if one uses the prediction accuracy values given in the paper [16]. We looked into these sequence differences, and found that our test dataset is indeed the latest version in the RCSB PDB. Therefore, we re-implemented the approach SCPRED by following the details presented in the paper [16] and tested on our test dataset. Experimental results showed that the prediction accuracies of SCPRED got further improved by 0.6% over those reported in [16].

### Secondary structure prediction

Every amino acid in a protein sequence can be predicted into one of the three secondary structural elements, H (helix), E (strand), and C (coil). It is a problem known as protein secondary structure prediction, and many computational approaches have been developed in the past decades to predict the 3-state secondary structure from protein sequences. In this study we chose PSIPRED [30], which predicts protein secondary structure based on the position specific scoring matrices generated by PSI-BLAST [31] and was shown to outperform other competing prediction methods [25]. For example, the protein 1E0G has a domain with amino acid sequence DSITYRVRKGDSLSSIAKRHGVNIKDVMRWNSDTANLQPGDKLTLFVK. If we submit this sequence to the PSIPRED 2.6 web server [32], the predicted secondary structure to be returned will be CCEEEEECCCCCHHHHHHHHCCCHHHHHHHCCCCCCCCCCCEEEEEEC.

Our method takes the predicted secondary structure sequence as input, but it is not tied to any specific tool for the secondary structure prediction. Any improved secondary structure prediction [33-35] would generally lead our method to higher prediction accuracy.

### Chaos game representation of predicted secondary structure

**Figure 1:**
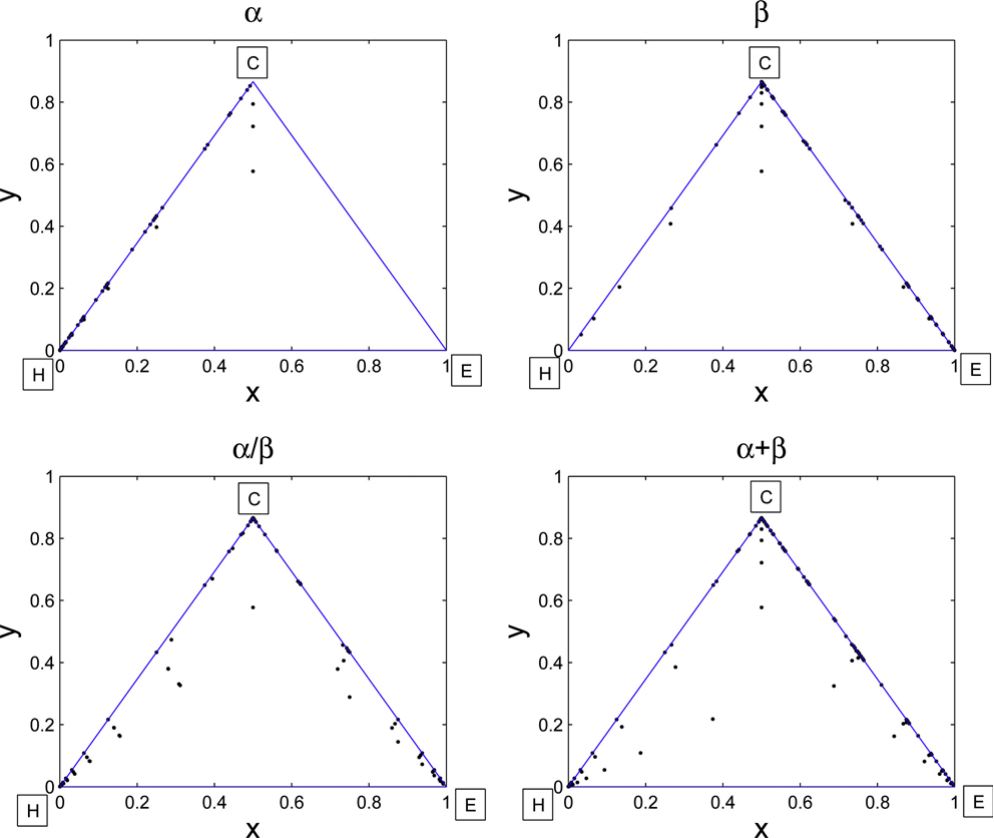
**The CGRs of predicted secondary structure for proteins from four structural classes**. The blue edges represents the sides of equilateral triangles and the black points represent the CGR points. The order of the black points (corresponding to the order in the predicted secondary structure) is saved, but not shown in the figure. The PDB IDs for four proteins are 1A6M (belonging to the *α *class), 1AJW (belonging to the *β *class), 1GQOV (belonging to the *α*/*β *class), and 1DEF (belonging to the *α *+ *β *class).

Here we develop a new set of features based on the *chaos game representation *(CGR) of secondary structure sequences. The CGR was initially developed to visualize DNA sequences [36], and later applied to protein sequences as well [17,37,38]. Given a secondary structure sequence, we start with a equilateral triangle with sides of unit length and each vertex associated with a distinct letter of H, E and C. For each letter of the given secondary structure sequence, we then plot a point inside the triangle as follows. The first point is placed half way between the center of the triangle and the vertex corresponding to the first letter of the secondary structure sequence, and the *i*-th point is then placed half way between the (*i *- 1)-th point and the vertex corresponding to the *i*-th letter. The obtained plot is then called the CGR of the secondary structure sequence. Figure 1 depicts the CGRs for four proteins, each belonging to a different structural class. It is very interesting to see that for proteins in the *α *and *β *classes, the plotted points tend to be distributed around the sides HC and EC, respectively. For proteins in the *α *+ *β *and *α*/*β *classes, however, the points lie around both sides HC and EC without preference.

**Figure 2:**
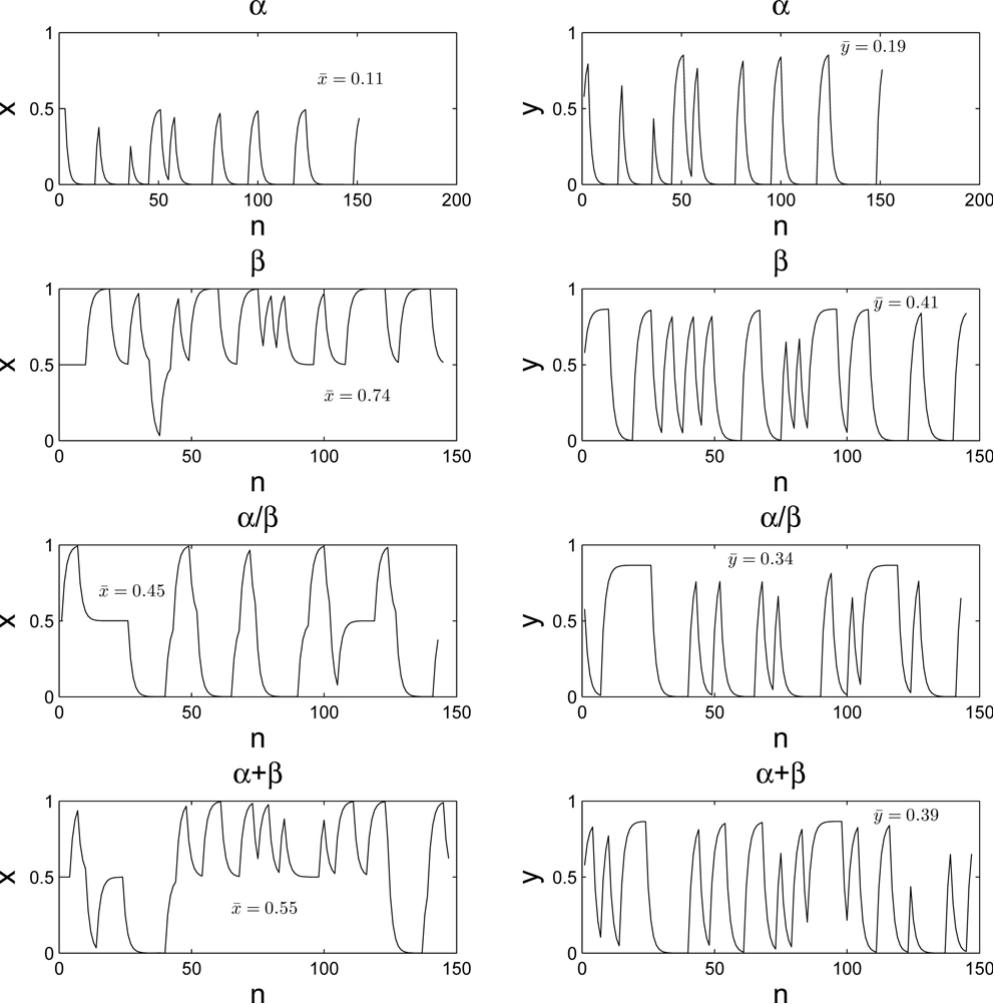
**Eight time series that represent the four CGRs in Figure 1**. Each panel in Figure 1 gives rise to two time series (*x*- and *y*-coordinates, respectively). As a result, we obtain eight time series for four CGRs.

Observe that every secondary structure sequence gives rise to a distinct (*x*, *y*)-coordinate sequence of the plotted points. Hence we can faithfully model a CGR plot as a combination of two time series, one composed of the *x*-coordinates and the other of the *y*-coordinates. For simplicity, we call them the *x*-time series and *y*-time series, respectively. As we can see from Figure 2, the average values of the *x*- and *y*-time series points for proteins in the *α*-class, denoted as  and  respectively, tend to be smaller than those for proteins in the other classes. Therefore, these two quantities will be used as the first two features in our feature set to be constructed.

One might think that the above two features (*i.e.*,  and ) are quite similar to computing the symbol frequencies in the input secondary structural sequence, except that one is performed in two dimension and the other in one dimension. Indeed, the features  and  take into account not only symbol frequencies but also symbol orders in the secondary structural sequence. For instance, if the input secondary structural sequence is permuted, the values of  and  will most likely change but the symbol frequencies will definitely not. It clearly demonstrates the advantage of features extracted from the chaos game representations of secondary structure sequences over those extracted directly from secondary structure sequences.

### Recurrence plot

*Recurrence plot *(RP) is a purely graphical tool originally proposed by Eckmann *et al. *[39] to detect patterns of recurrence in the data. Here, we use it to describe the natural time correlation information in a time series. Given a time series *z*_1_*z*_2 _⋯ *z*_*L *_of length *L*, we first embed it into the space *R*^*m *^of dimension *m *using a time delay *τ*. Let us define(1)

where *N*_*m *_= *L *- (*m *- 1)*τ*. Hence, we obtain *N*_*m *_vectors (*i.e.*, points) in the embedding space *R*^*m*^. While the values of *m *and *τ *have to be chosen appropriately based on nonlinear dynamical theory [40], *τ *is often set to be 1 in practice. Because an *α*-helix segment generally comprises at least three residues, we set *m *to be 3 in this study. We further construct a *distance matrix *(DM) of size *N*_*m *_× *N*_*m *_from the *N*_*m *_points, denoted as . Its elements *D*_*i*, *j *_are Euclidean distances between all pairs of points after scaled down by the maximum distance. As a result, all the element values of DM are located in the interval between 0 and 1, which allows the recurrence plots in different scales to be statistically compared [40]. Finally, we define a *recurrence matrix *(RM) by applying a *threshold ε *(namely radius) on the element values of DM. Formally, let  and(2)

where *H *is the *Heaviside function*; that is, *H*(*x*) = 0 if *x *< 0, and *H*(*x*) = 1 if *x *≥ 0.

**Figure 3:**
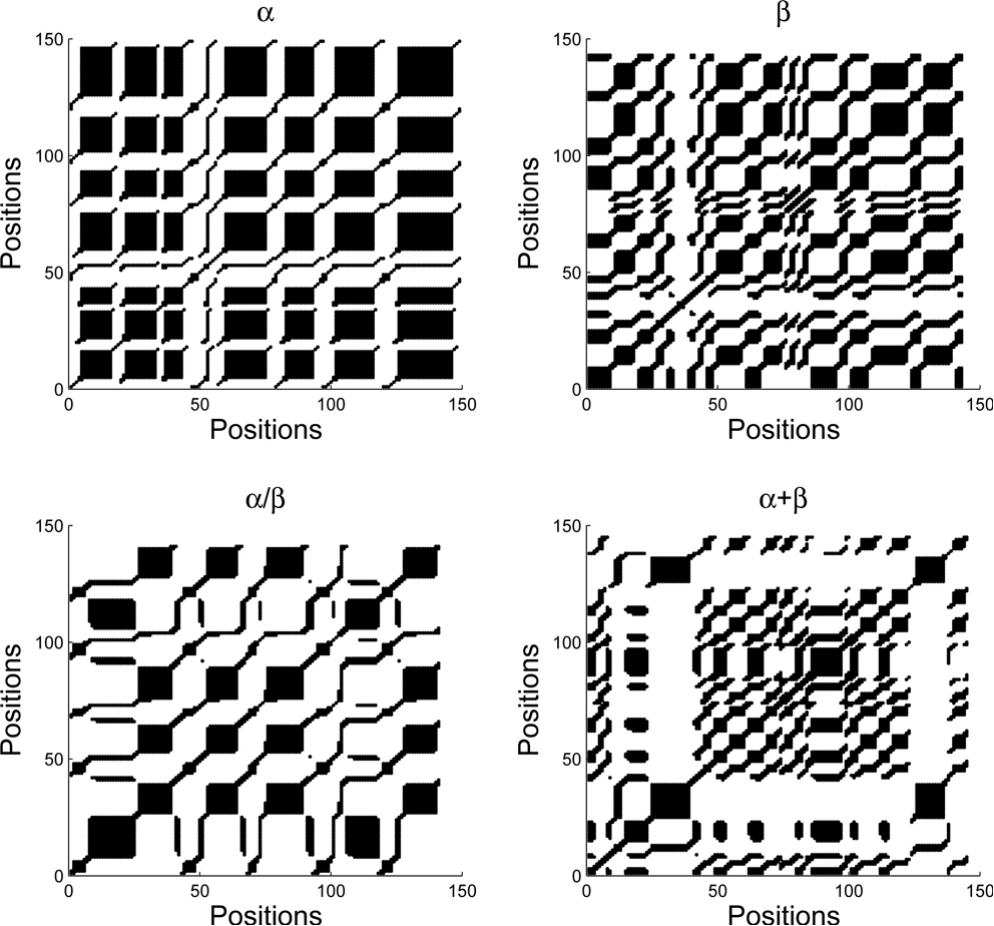
**The corresponding RPs for the four *x*-time series in Figure 2**. The parameters used are *m *= 3, *τ *= 1, and *ε *= 20%. Note that there is a black line along the main diagonal in the plots since a point always recurs with itself. Moreover, the points in the RP are symmetric with respect to the main diagonal line.

**Figure 4:**
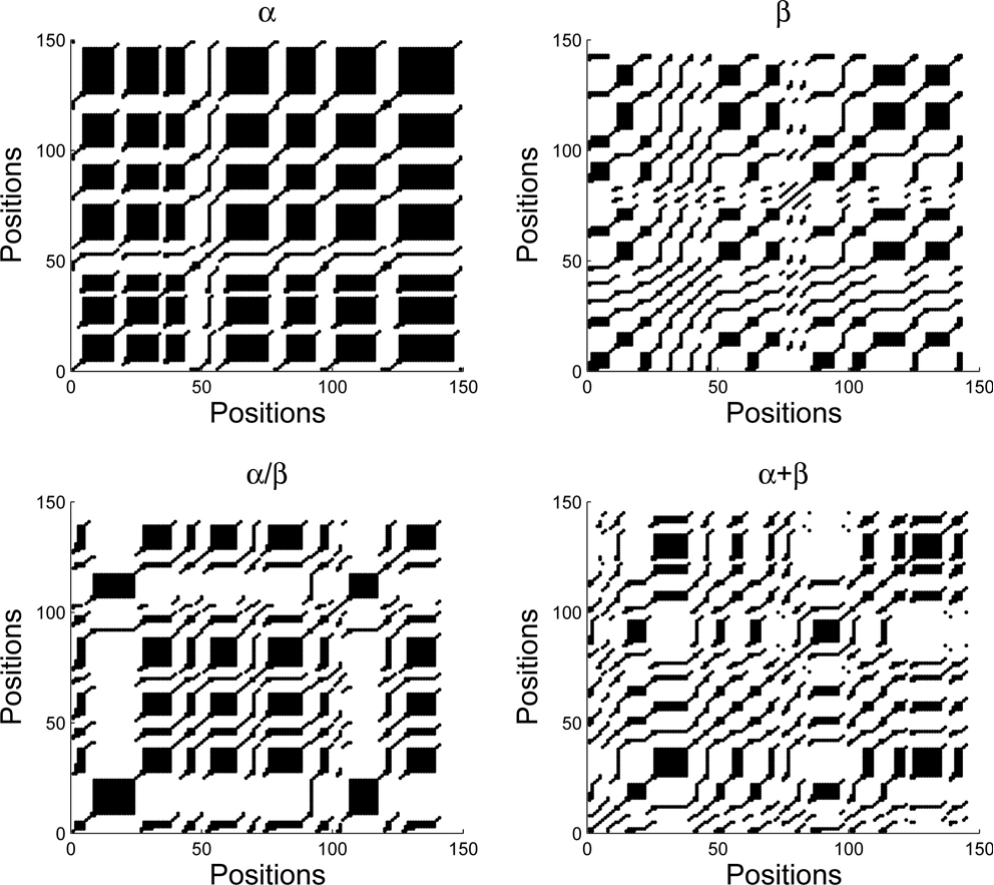
**The corresponding RPs for the four *y*-time series in Figure 2**. The parameters used are *m *= 3, *τ *= 1, and *ε *= 20%. Some interesting patterns can be seen to emerge from the plots, but it is not so easy to characterize them. In this study we chose the recurrence quantification analysis (RQA).

RP is simply a visualization of RM by plotting points on *i*-*j *plane for those elements in RM with values equal to 1. If *R*_*i*, *j*_(*ε*) = 1, we say the *j*-th point recurs with reference to the *i*-th point. For any 0 <*ε *< 1, the RP has always a black line along main diagonal since *R*_*i*, *i*_(*ε*) ≠ 1. Furthermore, the RP is symmetric with respect to the main diagonal as *R*_*i*, *j*_(*ε*) = *R*_*j*, *i*_(*ε*). For example, the RPs of the four *x*-time series (and *y*-time series) of Figure 2 are shown in Figure 3 (and Figure 4, respectively). It can be seen that *ε *is a crucial parameter in the construction of a RP. If *ε *is chosen too small, then there might leave only a few of recurrence points so that we can not learn any recurrence structure of the underlying time series. But if *ε *is too large, almost all the points will be enclosed in the neighbor of a point, thereby introducing a lot of structure artifacts. We will discuss the selection of the *ε *value later in this section.

### Recurrence quantification analysis

*Recurrence quantification analysis *(RQA) is a nonlinear technique used to quantify the information supplied by a recurrence plot [41,42]. In a previous study we applied the RQA to amino acid sequences for the prediction of protein structural classes [17]. Here we use it instead to analyze the predicted secondary structure sequences. Compared to 20 states (*i.e.*, bases) of amino acid sequence, the predicted secondary structure sequences have only three states (*i.e.*, H, E and C). By applying the recurrence quantification analysis, we obtain eight recurrence variables to characterize a predicted secondary structure sequences. The definitions of these eight recurrence variables are omitted here due to the page limit. Instead, they are provided in the Additional file 1 for readers' reference. These variables will be included into our set of features for the protein structural class prediction.

### *K*-string based information entropy

Given a predicted secondary structure sequence of length *L*, we call any substring *s*_1_*s*_2 _⋯ *s*_*K *_of length *K *a *K*-string, where each *s*_*i *_represents a letter in the set {H, E, C}. There are totally 3^*K *^distinct *K*-strings for any *K*. We denote the probability of the *K*-string *s*_1_*s*_2 _⋯ *s*_*K *_occurring in the given predicted secondary sequence by *p*(*s*_1_*s*_2 _⋯ *s*_*K*_). When *K *= 1, *p*(H), *p*(E), and *p*(C) are simply the respective probabilities of H, E and C occurring in the given predicted secondary structure sequence. The *K*-th order information entropy *I*_*K *_is hence calculated as(3)

where the first sum is over the set {H, E, C} and the second one is over all possible (*K *- 1)-strings. *p*(*s*|*s*_1_*s*_2 _⋯ *s*_*K*-1_) is the conditional probability of the letter *s *occurring after the (*K *- 1)-string *s*_1_*s*_2 _⋯ *s*_*K*-1 _in the given predicted secondary structure sequence. As argued in [18], *I*_*K *_can reflect certain local interactions (*i.e.*, correlations) along the secondary structure by using *K*-strings.

The two quantities, *p*(H) and *p*(E), are included in our feature set, and have been shown very helpful in improving the prediction of protein structural classes [16]. *p*(C) is not included as its value depends on *p*(H) and *p*(E) due to *p*(H) + *p*(E) + *p*(C) = 1. In addition, we include *I*_2_, *I*_3_, ..., *I*_*K *_into our feature set; however, the value of *K *remains to be determined. We will discuss this issue later in this section.

### Segment-based analysis

While proteins in the *α *+ *β *class and *α*/*β *class contain both *α *helices and *β *strands, they differ at least in two aspects. One is the directionality of *β *strands. The *β *strands are mainly antiparallel in the *α *+ *β *class but parallel in the *α*/*β *class. The other concerns the distributions of *α *helices and *β *strands. *α *helices and *β *strands are largely segregated in the *α *+ *β *class, but instead largely interspersed in the *α*/*β *class. While the exact distributions can only be known from the spatial arrangement of secondary structure segments, it is still reasonable to expect that they could be more or less inferred from their secondary structure sequences. To this end, we construct below three features from the secondary structure sequences characterizing the distributions of *α *helices and *β *strands, and hope that they can be used to differentiate between the *α *+ *β *class from *α*/*β *class.

As the first step of feature construction, we reduce a secondary structure sequence into a *segment *sequence, which is composed of *helix segments *and *strand segments *(denoted by *α *and *β*, respectively). Here, a helix segment refers to a continuous segment of all H symbols in the secondary structure sequence, and a similar definition is also applied to a strand or coil segment. Since at least three and two residues are generally required to form an *α *helix segment and an *β *strand segment respectively, we will ignore those helix and strand segments that do not meet this size requirement. Moreover, in order to focus on the arrangement of *α *helix and *β *strand segments, the coil segments are ignored as well. For example, given a secondary structure sequence, CCEECCCHHCCHHHHEEEHHHHCCCCCCECCEECCHHHCCEEEEEEC, its reduced segment sequence is *βαβαβαβ*, in which the *α *helices and *β *strands are largely interspersed, suggesting that the corresponding protein more likely belongs to the *α*/*β *class rather than *α *+ *β *class.

Let *p*_*t *_denote the probability of transitions between *α *and *β *segments in a segment sequence, which is essentially the relative frequency of the substring *αβ *or *βα *occurring in the segment sequence. Let  (respectively, ) denote the probability of two consecutive *α *(respectively, *β*) segments. Note that *p*_*t *_+  +  = 1; therefore, any probability can be deduced from the other two. In order to measure the degree of segment aggregation, we chose two of the above three probabilities to be included into our feature set. In our experimental study, *p*_*t *_and  are used. The third feature to be extracted is the probability of helix (or strand) segments occurring in a segment sequence, denoted by *p*(*α*) (or *p*(*β*)). Clearly, *p*(*α*) + *p*(*β*) = 1. *p*(*β*) is used in this study.

### Prediction assessment

As discussed above, we extract a set of 23+*K *- 1 features from the predicted secondary structure sequences. These feature vectors are fed into Fisher's discriminant algorithm [19] for the prediction of protein structural classes. Due to the page limit, the details of this algorithm is omitted. The prediction accuracy is measured by the proportion of proteins that are correctly predicted. The *jackknife test *is employed to evaluate our method. For more details about Fisher's discriminant algorithm and the prediction assessment, please refer to the Additional file 1.

### Selection of *ε *and *K*

**Figure 5:**
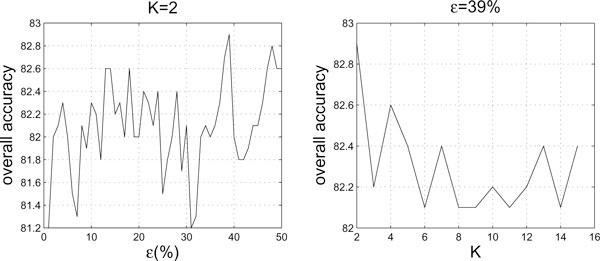
**The overall prediction accuracies of the dataset 25PDB with varying values of *ε *and *K***. When *K *= 2, *ε *ranges from 1% to 50% (left panel). When *ε *= 39%, *K *ranges between 2 and 15 (right panel).

As mentioned earlier, the value *ε *in RQA and the number *K *for *K*-string remain to be determined. Here, we determine their values by aiming to achieve the highest overall prediction accuracy as possible. For this purpose, a simple grid search strategy is adopted, where *ε *is allowed to take a value only between 1% to 50% and *K *only between 2 to 15. We use the dataset *25PDB *to compute the overall prediction accuracies for different combinations of *ε *and *K*. For example, when *K *= 2, the overall prediction accuracies for different values of *ε *are shown in the left panel of Figure 5. When *ε *= 39%, the overall prediction accuracies for different values of *K *are shown in the right panel of Figure 5.

By the above grid search, we found that the highest accuracy (83.0%) is obtained with the combination of *ε *= 48% and *K *= 14 (giving rise to 36 features) and the second highest accuracy (82.9%) is given by the combination of *ε *= 39% and *K *= 2 (giving rise to 24 features), which is only 0.1% lower. To gain such a negligible accuracy improvement, the former combination consumes a much larger amount of computer time and memory than the latter combination when calculating feature vectors of higher dimension (i.e., 36-dimension v.s. 24-dimension). Based on this observation, we chose *ε *= 39% and *K *= 2 in our experiments.

## Competing interests

The authors declare that they have no competing interests.

## Authors' contributions

JYY contributed to the conception and design of the study, downloaded the datasets, analyzed the results, has been involved in programming, drafting and revising the manuscript. ZLP has been involved in preparing the datasets, programming and discussion on the results. XC coordinated the study and has been involved in drafting and revising the manuscript. All authors read and approved the final manuscript.

## Supplementary Material

Additional file 1 The detailed descriptions about Recurrence quantification analysis, Fisher's discriminant algorithm and Prediction assessment can be found in this file. Click here for file
