# Supplementary material for: Prediction of protein structural classes for low-homology sequences based on predicted secondary structure
Source: BMC Bioinformatics. 2010 Jan 18;11(Suppl 1):S9. doi: 10.1186/1471-2105-11-S1-S9 (PMC3009544; doi:10.1186/1471-2105-11-S1-S9)
Supplement: Additional file 1 — The detailed descriptions about Recurrence quantification analysis, Fisher's discriminant algorithm and Prediction assessment can be found in this file. [file 1471-2105-11-S1-S9-S1.pdf]

Recurrence quantification analysis

Recurrence quantification analysis (RQA) is a nonlinear technique used to quantify the information supplied by a recurrence plot [1, 2]. In a previous study we applied the RQA to the prediction of protein structural classes directly from amino acid sequences [3]. Here we use it instead to analyze the predicted secondary structure sequences. Compared to 20 states (*i.e.*, bases) of amino acid sequence, the predicted secondary structure sequences have only three states (*i.e.*, H, E and C). In the following we briefly introduce the RQA techniques, where eight recurrence variables are defined to quantify a RP. These variables will be included into our set of features for protein structural class prediction. Because the RP is symmetric with respect to the main diagonal, the recurrence points considered in the following definitions will only comprise those in the upper triangle of a RP (excluding the main diagonal line as well).

The first recurrence variable is called *recurrence* (REC). It is a measure of the density of recurrence points in a RP, taking a value ranging from 0 (when there is no recurrent point) to 1 (when all points are recurrent). That is,

$$REC = \frac{\#\text{ recurrent points in upper triangle}}{N_m(N_m-1)/2}, \tag{1}$$

where # stands for counting the number of points.

The second recurrence variable is called *determinism* (DET). It measures the proportion of recurrent points that form diagonal line structures. Before evaluating this variable, we need to set the minimum number of recurrent points that a diagonal line segment requires. The commonly used number is 2, which is used in this study as well. Formally, we define determinism as

$$DET = \frac{\#\text{ recurrent points in diagonal lines}}{\#\text{ recurrent points}}. \tag{2}$$

The third recurrence variable is called *linemax* and denoted by L_{max}. It simply represents the length of the longest diagonal line segment in RP, and essentially inversely scales with the largest positive Lyapunov exponent [4]. Note that in general, the longer a time series, the longer diagonal line segments as well. In order to cancel the length influence of the time series (equal to the length of the corresponding protein amino acid sequence), we normalize the length of the longest diagonal line segment by dividing N_m. That is,

$$L_{max} = \frac{\text{length of longest diagonal line in RP}}{N_m}. \tag{3}$$

The fourth recurrence variable is *entropy* (ENT), which is the Shannon information entropy of the distribution probability of the length of the diagonal lines. That is,

$$ENT = - \sum_{k=L_{min},\ p(k)\neq 0}^{L_{max}} p(k) \log_2(p(k)), \tag{4}$$

where L_{min} is the minimum length of diagonal lines in RP and

$$p(k) = \frac{\#\text{ diagonal lines of length } k \text{ in RP}}{\#\text{ diagonal lines in RP}}. \tag{5}$$

The fifth recurrence variable is called *trend* (TND), which quantifies the stationarity degree of time series. It is calculated as the level that the *local recurrences* of diagonal lines fits their displacements from the main diagonal by least squares regression, where the *local recurrence* of a diagonal line refers to the proportion of points on the diagonal line that are the recurrence points. We would like to emphasize that the variable *recurrence* is defined on the whole upper triangle of RP while the *local recurrence* is instead defined only on a certain diagonal line of RP.

The remaining three variables are defined based on the vertical line structure. The sixth recurrence variable is called *laminarity* (LAM). It is analogous to DET, but calculated using recurrence points forming vertical line structures. That is,

$$LAM = \frac{\#\text{recurrent points in vertical lines}}{\#\text{recurrent points}}. \tag{6}$$

The seventh variable, called *trapping time* (TT), is the *normalized* average length of vertical line structures (i.e., average length of vertical line structures divided by N_m). The eighth recurrence variable is the *maximum normalized length of the vertical lines* in RP, which is analogous to the definition of L_{max} and denoted by V_{max}.

Fisher's discriminant algorithm

As discussed above, we extract a set of $23{+}K - 1$ features from the predicted secondary structure sequences. Therefore, each protein is represented by a $(23{+}K - 1)$-D vector. The value of K is chosen in order to maximize the overall prediction accuracy. Various classification algorithms have been employed in the literature to predict protein structural classes, including support vector machines [5], logistic regression [6, 7], fuzzy clustering [8] and fuzzy K nearest neighbors [9]. In this study, we chose a simple yet

powerful Fisher's discriminant algorithm [10].

Fisher's discriminant algorithm works as follows. It first builds a classifier in the parameter space for a training dataset. A training set $H = \{x_1, x_2, \cdots, x_n\}$ is given to contain training vectors from two classes. There are n_1 training vectors from one class forming a subset H_1 and n_2 training vectors from another class forming a subset H_2. Hence, $n_1 + n_2 = n$ and $H = H_1 \cup H_2$. Assume that each x_i is a m-dimension vector. Then, a parameter vector $\mathbf{w} = (w_1, w_2, \cdots, w_m)^T$ is estimated such that it allows as many training vectors as possible to be accurately predicted. Specifically,

$$\mathbf{m}_j = \frac{1}{n_j} \sum_{\mathbf{x_i} \in H_j} \mathbf{x_i}, \quad j = 1, 2, \tag{7}$$

$$\mathbf{S}_j = \sum_{\mathbf{x_i} \in H_j} (\mathbf{x_i} - \mathbf{m_j})(\mathbf{x_i} - \mathbf{m_j})^T, \qquad j = 1, 2, \tag{8}$$

$$\mathbf{S}_w = \mathbf{S}_1 + \mathbf{S}_2, \tag{9}$$

and

$$\mathbf{w} = \mathbf{S}_w^{-1}(\mathbf{m_1} - \mathbf{m_2}), \tag{10}$$

By Fisher's discriminant rule, $\mathbf{x}$ is hence assigned to the class of H_1 if
$dist = (\mathbf{m_1} - \mathbf{m_2})^T \mathbf{S}_w^{-1}[\mathbf{x} - \frac{1}{2}(\mathbf{m_1} + \mathbf{m_2})] > 0$ and to the class of H_2 otherwise.

The above algorithm is designed for a two-class problem, i.e., it provides a binary classifier. We could transform a four-class problem of protein structural classes prediction into six two-class problems, namely, α-vs-β, α-vs-$(\alpha + \beta)$, α-vs-(α/β), β-vs-$(\alpha + \beta)$, β-vs-(α/β) and $(\alpha + \beta)$-vs-(α/β). That is to say, six binary classifiers are trained and each classifier gives one vote for its preferred class. The final prediction will be the class with the most votes. Once a tie happens, we calculate a weight as the sum of the absolute value of *dist* over all votes in the tied cases and the final prediction is hence the class with the largest weight.

Prediction assessment

The *jackknife test* is a rigorous and objective statistical test that can always yield a unique result for a given test dataset [11]. Therefore, it is often used to examine the power of a new predictor. In this paper, we also use it to evaluate our method, where proteins are singled out from the dataset one by one as a testing protein and the predictor is trained by the remaining proteins. In this sense, jackknife test is also called the *leave-one-out* test. The prediction accuracies are then measured by the following formulae.

$$\text{accuracy}_\alpha = \frac{a}{N_a}, \quad \text{accuracy}_\beta = \frac{b}{N_b}, \quad \text{accuracy}_{\alpha+\beta} = \frac{c}{N_c}, \quad \text{accuracy}_{\alpha/\beta} = \frac{d}{N_d}, \tag{11}$$

and

$$\text{accuracy}_{\text{overall}} = \frac{o}{N_o}. \tag{12}$$

In the above, a, b, c and d denote the number of correctly predicted proteins in $\alpha, \beta, \alpha+\beta$ and α/β classes, respectively. N_a, N_b, N_c and N_d denote the total number of proteins in $\alpha, \beta, \alpha+\beta$ and α/β classes, respectively. Furthermore, $o = a + b + c + d$ and $N_o = N_a + N_b + N_c + N_d$.

References

1. Zbilut JP, Webber CLJ: **Embeddings and delays as derived from quantification of recurrence plots**. *Phys Lett A* 1992, **171**:199–203.
2. Webber CLJ, Zbilut JP: **Dynamical assessment of physiological systems and states using recurrence plot strategies**. *J Appl Physiol* 1994, **76**:965–973.
3. Yang JY, Peng ZL, Yu ZG, Zhang RJ, Anh V, Wang D: **Prediction of protein structural classes by recurrence quantification analysis based on chaos game representation**. *J Theor Biol* 2009, **257**:618–626.
4. Eckmann JP, Kamphorst SO, Ruelle D: **Recurrence plots of dynamical systems**. *Europhys Lett* 1987, **4**:973–977.
5. Kurgan L, Cios K, Chen K: **SCPRED: Accurate prediction of protein structural class for sequences of twilight-zone similarity with predicting sequences**. *BMC Bioinformatics* 2008, **9**:226.
6. Kedarisetti KD, Kurgan LA, Dick S: **Classifier ensembles for protein structural class prediction with varying homology**. *Biochem Biophys Res Commun* 2006, **348**:981–988.
7. Kurgan LA, Homaeian L: **Prediction of structural classes for protein sequences and domains–Impact of prediction algorithms, sequence representation and homology, and test procedures on accuracy**. *Pattern Recogn* 2006, **39**:2323–2343.
8. Shen HB, Yang J, Liu XJ, Chou KC: **Using supervised fuzzy clustering to predict protein structural classes**. *Biochem Biophys Res Commun* 2005, **334**:577–581.
9. Zhang TL, Ding YS, Chou KC: **Prediction protein structural classes with pseudo-amino acid composition: Approximate entropy and hydrophobicity pattern**. *J Theor Biol* 2008, **250**:186–193.
10. Duda RO, Hart PE, Stork DG: *Pattern Classification.* New York: John Wiley & Sons 2001.
11. Chou KC, Shen HB: **Cell-PLoc: A package of web-servers for predicting subcellular localization of proteins in various organisms**. *Nature Protocols* 2008, **3**:153–162.
